# Vitamin D and calcium status in HYDRIA, Greece: associations with dietary and supplemental sources

**DOI:** 10.1007/s00394-026-03962-4

**Published:** 2026-04-10

**Authors:** Georgia Vourli, Eleni Peppa, Eleni Maria Papatesta, Maria Kritikou, Antonia Trichopoulou

**Affiliations:** Center for Public Health Research and Education, Academy of Athens, Athens, Greece

**Keywords:** Vitamin D, Calcium, Nutrition, Supplements

## Abstract

**Purpose:**

Serum vitamin D and calcium levels have been associated with health outcomes, while their deficiency, especially of vitamin D, is prevalent worldwide. This study aimed to estimate serum vitamin D and calcium levels in Greece, describe dietary intakes and supplement use, and identify characteristics associated with their status.

**Methods:**

The study included 685 HYDRIA participants aged  ≥ 54 years. Associations of blood levels with demographic, lifestyle, and nutritional data were assessed via linear and logistic regression models.

**Results:**

Mean serum vitamin D was 22.7 ng/ml (95% CI 21.6–23.8), significantly lower in women than men (21.1 vs. 24.6 ng/ml; *p* = 0.008). Vitamin D deficiency (< 20 ng/ml) affected 54.3% of women and 39.4% of men. Dietary intake was similar between sexes, though supplement use was higher among women (11.6% vs. 1.2%; *p* = 0.002). Vitamin D levels were significantly associated with supplement use (8.31 ng/ml; *p* < 0.001), high education (2.96; *p* = 0.016), sex (− 4.04 ng/ml in women; *p* = 0.001) and age, with levels rising at younger ages and plateauing or declining at older ages. For calcium, women had lower intake than men (1053.1 vs. 860.4 mg/day; *p* < 0.001), and were the only group using calcium supplements (12.1%). Only calcium adjusted for a 2000 kcal diet was significantly associated with calcium blood levels (*p* = 0.024).

**Conclusion:**

These findings highlight a high prevalence of vitamin D deficiency, particularly among women. Apart from sex, supplement use was the only significant predictor of higher vitamin D levels, suggesting diet alone may be insufficient. Calcium dietary intake was lower in women. These results emphasize the importance of targeted interventions, such as personalized guidance and awareness campaigns. Further research is needed to quantify the impact of vitamin D and calcium deficiency on human health.

**Supplementary Information:**

The online version contains supplementary material available at 10.1007/s00394-026-03962-4.

## Introduction

Vitamin D can be obtained in the human organism through two separate pathways: sunlight exposure, or its intake either from food or from supplements [6, 17]. Exposure to the sunlight represents the main way of producing vitamin D, since there are very limited dietary sources. Once synthesized or ingested, vitamin D is metabolized in the liver to 25-hydroxyvitamin D (25(OH)D), the primary indicator of vitamin D status in clinical settings, and then to its active form, 1,25-dihydroxyvitamin D (1,25(OH)_2_D) [6, 17].

Unlike vitamin D, calcium is a mineral mainly obtained through the diet, or alternatively from supplements or medication. In Greece, as in most European countries, the main calcium dietary sources are dairy products, cereals and their products, as well as some vegetables [47]. The efficiency of the reabsorption of renal calcium and of intestinal calcium and phosphorus is increased in the presence of 1,25(OH)_2_D, while 1,25(OH)_2_D production is tightly regulated by plasma parathyroid hormone levels and serum calcium and phosphorus levels [6, 8, 17].

Vitamin D deficiency has many health effects and it is therefore a global health concern. Its role in skeletal health is well established, with deficiency contributing to rickets in infants and children and osteomalacia in adults, although such manifestations are now relatively uncommon in developed countries [20, 39]. Beyond bone metabolism, low serum vitamin D levels have been associated with an increased risk of musculoskeletal injuries, with muscle strength impairment, with a higher incidence of hip fractures in older adults and with fertility regulation and pregnancy outcomes [5, 16, 28, 38]. It is also recognized that vitamin D plays an important role to the immune system regulation and to neurocognitive health [21, 41].

In recent years vitamin D deficiency has become the most common nutritional deficiency worldwide with more than 1 billion people having inadequate serum vitamin D concentrations [18, 46].

Calcium intake has likewise been associated with numerous health outcomes. Beyond its importance in bone health, studies have reported protective effects of adequate calcium intake and supplementation on maternal health, particularly in reducing the risk of preeclampsia and preterm birth [15, 23, 42, 43].

Combined supplementation of vitamin D and calcium has been shown in some studies to reduce fracture risk and all-cause mortality in postmenopausal women, although other studies report no effect or adverse outcomes such as increased risk of nephrolithiasis [2, 25, 33]. Recent evidence also indicates that vitamin D supplementation does not significantly reduce major cardiovascular events [34], underscoring the complexity of interpreting the health effects of these nutrients.

Despite the extensive research on vitamin D and calcium, there is limited population-level data in Greece. Given the importance of their status and considering the aging of the population, epidemiological data are lacking. The aim of this study is to estimate dietary vitamin D and calcium intake, supplements and/or medication use, as well as serum blood levels in a representative sample of the ≥ 54-years-old Greek population. Moreover, we aim to examine the factors affecting vitamin D and calcium levels and the relationship between them.

## Methods

HYDRIA is a national survey that was performed in Greece, from June 2013 to December 2014 and includes 4011 adult participants. Data were collected respecting the guidelines of the Helsinki Declaration and the national data protection legislation. HYDRIA was designed as a two-stage, stratified, random sample survey, that when appropriately weighted and analyzed the findings are representative of the adult Greek population. The data collection was performed following the recommendations of the European Health Examination Survey (EHES) [11]. The design of the survey allows the inter-country comparisons in accordance with the European Food Safety Authority (EFSA) requirement [9]. Details regarding HYDRIA’s study tools and methods have been previously published [31, 32]. The blood samples were analyzed by “Bioiatriki” Healthcare Group. For calcium, a colorimetric assay using NM-BAPTA was used, and for vitamin D, an ElectroChemiLuminescence Immunoassay (ECLIA) was employed. To assess dietary intake, two non-consecutive 24-h dietary recalls (24HDR) were collected from each participant. Supplement use was also recorded.

HYDRIA participants aged 54 years old or older at the time of the study were selected to undergo vitamin D blood level measurement, and these individuals constitute the sample analyzed here (*N* = 685). For these individuals, calcium was also available.

### Statistical analysis

For the continuous variables, vitamin D and calcium mean amounts intake from diet are presented unadjusted, and in few cases adjusted for a 2000 kcal diet, as well. For the corresponding levels found in blood, mean levels are provided. In case of categorical variable, as the use of drugs/supplements of vitamin D and calcium, percentages are presented. All descriptive analysis is performed by subgroups according to individual’s characteristics (sex, age group, smoking status, community setting, occupation, educational level, BMI category). In all cases, 95% Confidence Intervals (95% CI) are presented along with the point estimates. All comparisons are performed using univariable models, linear in case of comparisons of a continuous outcome (e.g. vitamin D and calcium blood levels) or logistic when percentages between subgroups are compared (e.g. the use of supplements).

Linear regression was used to identify factors that have a significant effect on vitamin D and calcium blood levels. In the case of vitamin D, it was categorized in three groups using the cut-offs of 30 and 20ng/ml, i.e. > 30ng/ml, 20.1–30ng/ml, ≤ 20ng/ml, suggesting vitamin D deficiency ≤ 20ng/ml and vitamin D insufficiency 20.1–30ng/ml [19]. An ordinal regression model for this new three-level variable was fitted. In multivariable analyses, all individual’s characteristics referred above were included as potential risk factors. For calcium, the age was entered as a categorical covariate, since the effect of the continuous variable was very small and not significant.

To account for the HYDRIA survey’s sampling design and for deviations from the Greek population according to the 2011 census, weighting factors were applied in all analysis. To adjust for non-response, as a sub-sample of those aged above 54 years old had not measured blood D levels, we applied the inverse probability weighting method. Weights were the reciprocal of the response probabilities estimated through weighted logistic regression. Final weights were the product of study design and non-response weights.

All analysis was performed at α = 0.05 level of significance, using the Stata software (Stata/SE 11.2, StataCorp LP).

## Results

**Fig. 1:**
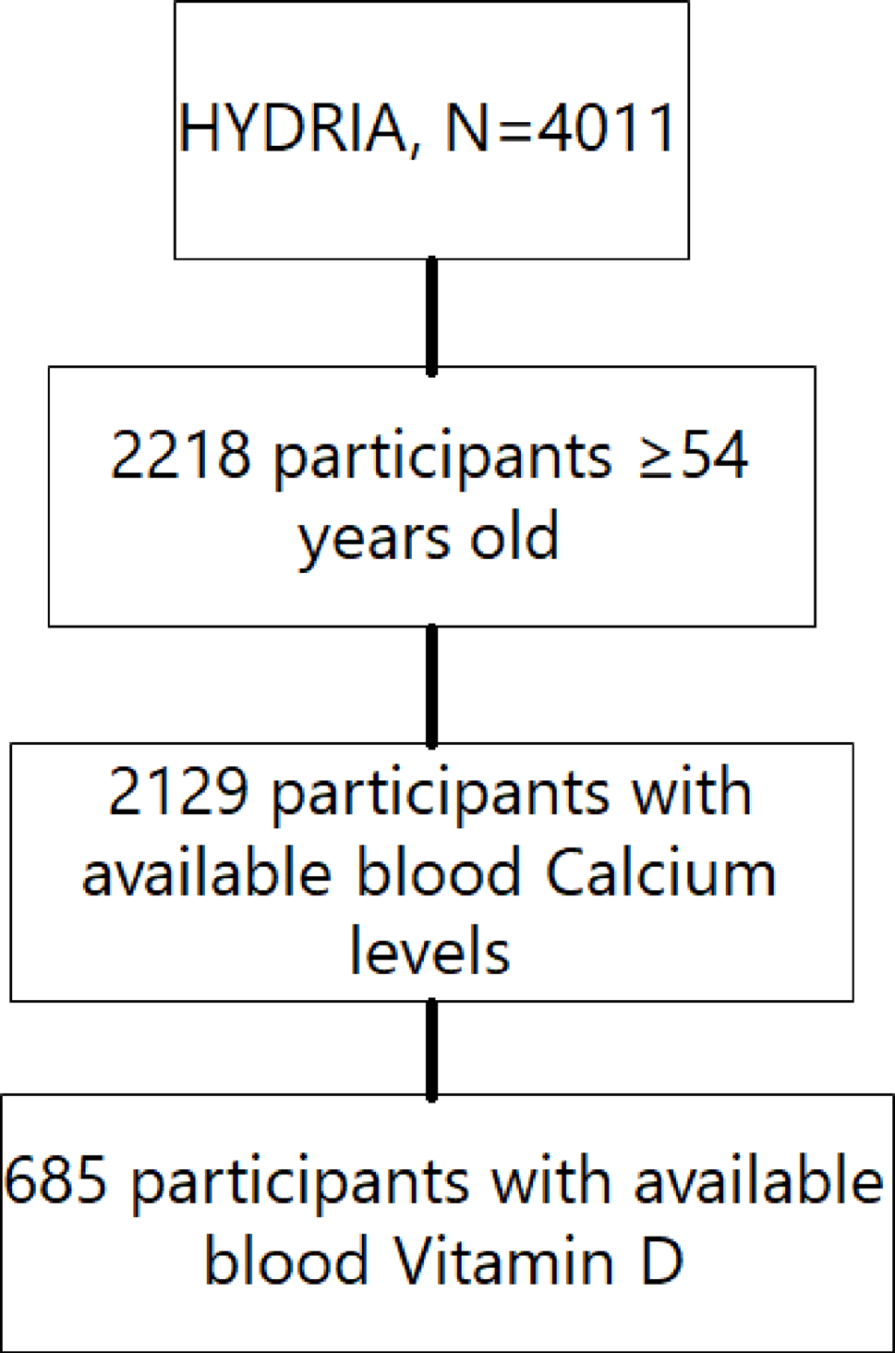
Flowchart for the sample selection procedure

The final sample included 685 HYDRIA participants (Fig. 1). Men and women were similarly represented in the sample; 44.0% of them are never smokers, while only 15.1% have a normal weight.

### Vitamin D

Overall, mean blood levels of vitamin D were 22.7ng/mL (95% CI 21.6–23.8) and it was significantly lower in women compared to men (21.1ng/mL (95% CI 19.6–22.7) versus 24.6ng/mL (95% CI 22.8–26.4), respectively; *p* = 0.008). Vitamin D deficiency was observed in 54.3% of women and 39.4% of men. Intake from diet was similar in men and women (2.0 vs. 2.1; *p* = 0.764), however the use of vitamin D supplements was higher among women (11.6% vs. 1.2% in men; *p* = 0.002) (Table 1).

Older adults aged ≥ 75 years exhibited the lowest mean vitamin D levels (20.2 ng/mL) and the highest deficiency prevalence (60.9%). Participants aged 54–64 years showed the lowest insufficiency (72.1%), whereas insufficiency reached 83.9% among those aged  ≥ 75 years.

**Fig. 2:**
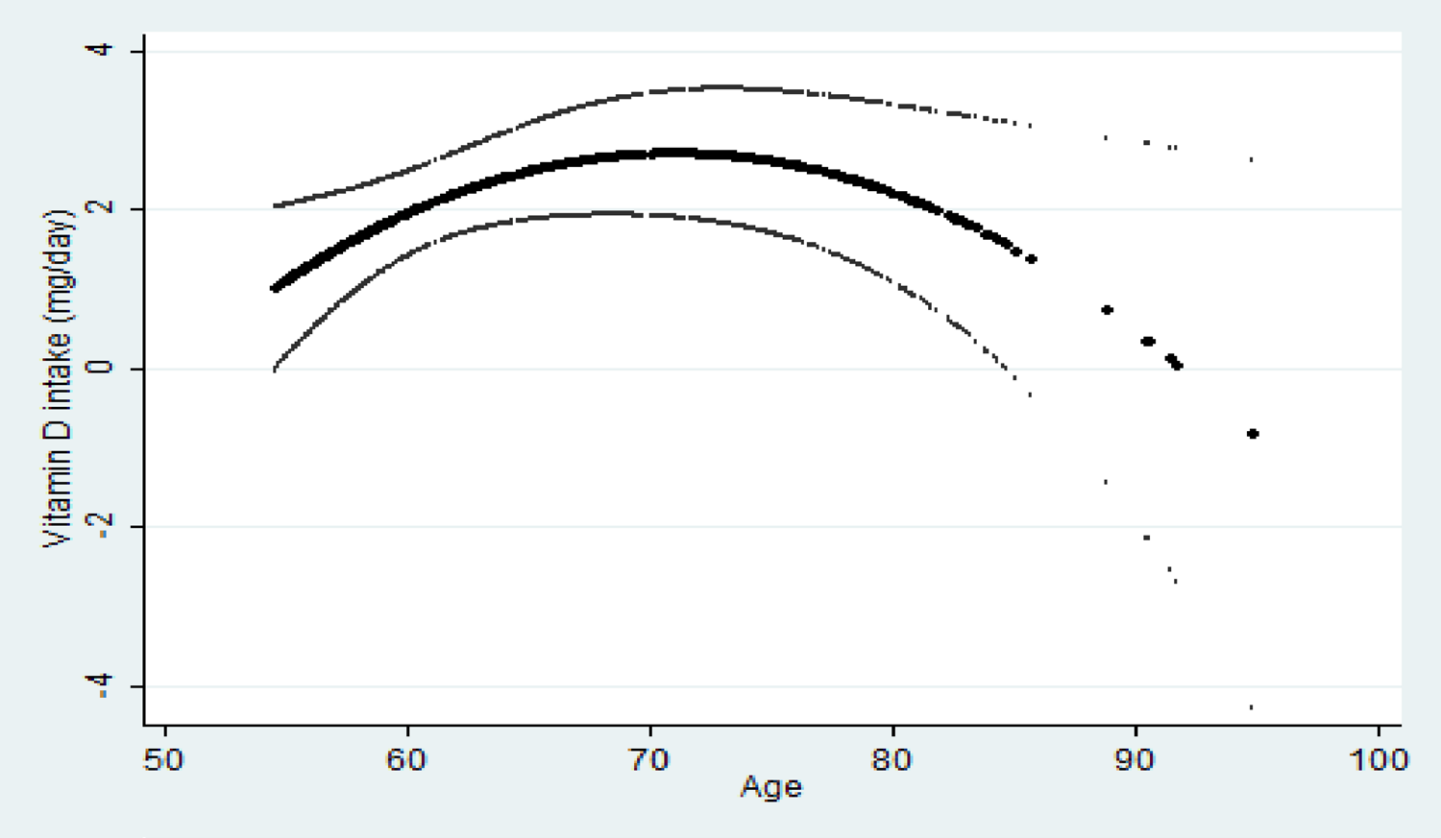
Predicted vitamin D intake as a function of age, along with the corresponding 95% confidence interval

The use of vitamin D drugs or supplements was less prevalent among individuals older than 75 years old compared to those aged 54–64 and 65–74 years old participants, however not significantly. Women who used vitamin D drugs or supplements had significantly lower vitamin D intake from their diet (*p* = 0.006). A significant inverse U-shaped relationship was observed between dietary vitamin D intake and age, as indicated by a linear regression model including age in quadratic form. This relationship is illustrated in Fig. 2, which presents predicted vitamin D intake against age, with the corresponding 95% confidence interval. Unemployed participants tended to have significantly lower dietary vitamin D intake compared to employed individuals (*p* = 0.051), however this association was not retained after adjusting for sex and age (*p* = 0.093). Participants with increased BMI, both overweight and obese had higher dietary vitamin D intake, compared to those with a BMI that indicated normal weight (*p* = 0.004), however obese participants tended to have lower blood vitamin D (*p* = 0.052). According to univariable linear regression analysis, only vitamin D supplements (6.81ng/mL increase; 95% CI 4.27–9.34; *p* < 0.001), sex (− 3.44ng/mL decrease for women; 95% CI − 5.95 to − 0.93; *p* = 0.010) and high versus low education (3.91 ng/mL increase; 95% CI 1.64–6.17; *p* = 0.001), were associated with the vitamin D blood levels (Supplement 1).

Vitamin D supplements use, sex, age (entered as a continuous variable, along with age squared) and educational level were independent prognostic factors for vitamin D blood levels. Supplement or medication use was the strongest predictor of higher vitamin D blood concentrations, being associated with an average increase of 8.31ng/mL (95% CI 5.65–10.98; *p* < 0.001). The association of Vitamin D blood with age followed a non-linear pattern, with levels increasing at younger ages and plateauing or declining at older ages (Supplement 2). Women had significantly lower vitamin D blood levels than men (− 4.04ng/mL; 95% CI − 6.46 to − 1.62; *p* = 0.001). Higher educational attainment was also associated with higher vitamin D blood levels, particularly among those with high education (2.96 ng/mL; 95% CI 0.56–5.37; *p* = 0.016) (Table 2A).

When considering vitamin D adequacy categories (> 30 ng/ml, 20.1–30 ng/ml, ≤ 20ng/ml), individuals taking supplements had 84% lower odds of being in a worse category (OR = 0.16; 95% CI 0.08–0.30; *p* < 0.001). Women had higher odds of worse vitamin D status compared to men (OR = 2.40; 95% CI 1.46–3.97; *p* = 0.001). Increasing age was also associated with greater severity of vitamin D deficiency (OR per 1 year increase = 1.03; 95% CI 1.01–1.06; *p* = 0.011) (Table 2B).

Dietary vitamin D intake, either energy-adjusted (per 2000 kcal) or unadjusted, was not significantly associated with vitamin D blood levels.

### Calcium

In this sample, none of the male participants used calcium drugs or supplements, whereas 12.1% of women reported such use. Women had, on average, a lower absolute dietary calcium intake (1053.1 mg/day in men vs. 860.4 mg/day in women; *p* < 0.001) although, however, when adjusted for energy intake, women consumed proportionally more calcium (per 2000 kcal: 1095.7 mg/day for men versus 1298.0 mg/day for women, *p* = 0.001) (Table 3). Overall, 13.4% (95% CI 7.3–19.5%) of women reported use of supplements, either vitamin D or calcium.

Only dietary calcium intake adjusted to a 2000 kcal energy intake was significantly associated with Calcium blood levels (for 1000 mg change: 0.011 mg/dl (95% CI 0.01–0.20), *p* = 0.024) (Table 4).

**Table 1:** Vitamin D supplement use, mean dietary intake, and mean blood levels among adults  ≥ 54 years, including prevalence of severe (≤ 20 ng/mL) and mild/severe (≤  30 ng/mL) deficiency

	Vitamin D intake		Vitamin D blood levels		
	Use of vitamin D supplements or medicines % (95% CI)	Vitamin D from diet(mg/day) (mean (95% CI))	Vitamin D levels in blood (ng/ml) (mean (95% CI))	Vitamin D deficiency (Vitamin D ≤ 20ng/ml) % (95% CI)	vitamin D insufficiency (Vitamin D ≤ 30ng/ml) % (95% CI)
Overall	6.8 (3.7–9.9)	2.1 (1.6–2.5)	22.7 (21.6–23.8)	47.5 (41.7–53.3)	75.7 (71.4–80.0)
*Sex*					
Male (*N* = 343)	1.2 (− 0.4–2.8)	2.0 (1.2–2.7)	24.6 (22.8–26.4)	39.4 (30.8–48.0)	69.3 (61.6–76.9)
Female (*N* = 342)	11.6 (6.2–17.0)	2.1 (1.4–2.9)	21.1 (19.6–22.7)	54.3 (46.2–62.4)	81.2 (75.2–87.2)
*Age groups*					
55–64 years (*N* = 310)	7.4 (3.0–11.9)	1.7 (1.1–2.3)	23.6 (22.0–25.2)	42.7 (33.6–51.8)	72.1 (65.6–78.6)
65–74 years (*N* = 227)	7.8 (1.9–13.6)	2.7 (1.8–3.7)	22.8 (20.7–24.8)	47.7 (37.8–57.6)	77.4 (69.4–85.5)
75 + years (*N* = 133)	3.8 (− 0.6–8.1)	2.1 (0.4–3.9)	20.2 (17.1–23.2)	60.9 (48.8–73.1)	83.9 (73.4–94.3)
*Occupation*					
Employed (*N* = 99)	5.6 (− 1.3–12.6)	2.4 (0.8–3.9)	22.3 (19.5–25.0)	46.7 (30.5–62.9)	78.2 (66.9–89.5)
Unemployed (*N* = 28)	4.3 (2.7–6.0)	0.5 (0.3–0.7)	27.1 (23.2–31.1)	40.0 (21.8–58.1)	50.8 (30.6–71.1)
Pensioner (*N* = 465)	6.6 (3.0–10.1)	2.2 (1.5–2.9)	23.2 (22.0–24.5)	44.9 (39.0- 50.8)	76.4 (72.1–80.7)
House-keeping (*N* = 93)	10.1 (− 0.6–20.9)	2.1 (0.9–3.2)	20.1 (17.6–22.6)	58.2 (44.7–71.6)	81.7 (70.5–92.8)
*Educational level*					
Low (*N* = 345)	7.4 (3.5–11.3)	2.2 (1.5–3.0)	21.6 (20.2–23.0)	51.1 (43.6–58.6)	80.5 (75.4–85.6)
Intermediate (*N* = 176)	6.7 (0.4–13.0)	1.2 (0.8–1.7)	24.1 (22.1–26.2)	45.0 (35.7–54.3)	69.3 (60.1–78.5)
High (*N* = 164)	4.6 (1.3–7.9)	2.4 (0.6–4.2)	25.5 (23.6–27.5)	34.7 (26.3–43.1)	63.8 (55.1–72.6)
*Smoking*					
Daily smokers (*N* = 140)	7.2 (− 1.0–15.4)	1.7 (0.6–2.7)	23.3 (20.8–25.8)	49.0 (37.6–60.5)	72.1 (61.0–83.3)
Occasional smokers (*N* = 23)	6.6 (− 5.6–18.8)	2.4 (0.8- 4.0)	22.1 (18.7–25.6)	34.3 (11.1–57.5)	88.1 (75.5–100.7)
Former smokers (*N* = 215)	5.3 (− 0.6–11.1)	2.5 (1.4–3.7)	24.0 (22.1–25.8)	41.7 (30.4–53.0)	71.8 (63.5–80.1)
Non smokers (*N* = 294)	8.0 (3.5–12.4)	1.9 (1.1–2.8)	21.7 (19.8–23.5)	51.0 (42.2–59.8)	79.5 (72.6–86.4)
*Urban level*					
urban (*N* = 385)	6.7 (2.6–10.8)	1.8 (1.3–2.3)	23.0 (21.6–24.4)	46.8 (40.1–53.6)	75.1 (69.7–80.5)
semi-urban (*N* = 150)	3.0 (− 0.2–6.3)	2.0 (0.8–3.2)	22.9 (20.3–25.5)	41.8 (25.9–57.7)	77.1 (66.6–87.7)
rural (*N* = 150)	8.9 (2.3–15.5)	2.6 (1.4–3.8)	22.1 (19.9–24.3)	51.4 (39.4–63.4)	76.1 (67.4–84.8)
*BMI*					
< 25 (*N* = 98)	9.5 (− 0.1–19.2)	0.8 (0.4–1.3)	24.6 (21.5–27.8)	44.1 (30.3–58.0)	61.9 (46.8–77.1)
25–29.9 (*N* = 278)	7.1 (3.2–11.0)	2.2 (1.4–2.9)	23.9 (22.0–25.7)	43.1 (34.9–51.3)	72.7 (64.8–80.5)
≥ 30 (*N* = 275)	5.8 (0.6–11.0)	2.5 (1.5–3.4)	21.2 (19.8–22.6)	52.1 (42.8–61.4)	83.4 (77.7–89.0)

Estimates account for sampling weights and complex survey design to ensure representativeness of the adult population. Mean values presented with 95% confidence intervals

**Table 2:** Final multivariable models: (A) linear regression for continuous blood vitamin D levels, and (B) ordinal logistic regression for vitamin D status categorized as normal (> 30 ng/mL), mild deficiency (20–30 ng/mL), and severe deficiency (< 20 ng/mL)

(A) Linear regression	Coefficient (95% CI)	*p*-value
Covariate		
Non-diet vitamin D intake (yes vs. no)	8.31 (5.65 to 10.98)	< 0.001
Age	1.57 (0.00 to 3.14)	0.050
Age^2^	– 0.01 (– 0.02 to 0.00)	0.034
Sex (Female vs. Male)	– 4.04 (– 6.46 to – 1.62)	0.001
Educational level		
Intermediate versus low	2.03 (– 0.35 to 4.42)	0.094
High versus low	2.96 (0.56 to 5.37)	0.016

(B) Ordinal logistic regression	Odds ratio (95% CI)	*p*-value
Non-diet vitamin D intake (yes vs. no)	0.16 (0.08 to 0.31)	< 0.001
Sex (Female vs. Male)	2.32 (1.42 to 3.79)	0.001
Age (years)	1.03 (1.01 to 1.06)	0.011

Models adjusted for all covariates listed. Coefficients in (A) indicate mean changes in vitamin D levels for each category change in the covariate; odds ratios in (B) reflect odds of greater deficiency category. Estimates account for sampling weights and complex survey design, along with non-response weights to ensure representativeness of the adult population

**Table 3:** Calcium supplement use, mean dietary intake, and mean blood levels among adults  ≥ 54 years

	Calcium intake		Calcium blood levels
	Percentage (%) of participants who use calcium supplements or medicines (95% CI)	Calcium from diet (mg/day, mean (95% CI))	Calcium levels in blood (mg/dl) (mean (95% CI))
Overall	6.6 (3.4–9.7)	948.1 (897.8–989.5)	9.5 (9.5–9.6)
*Sex*			
Male (*N* = 343)	0.0 (0.0–0.0)	1053.1 (979.6–1126.7)	9.5 (9.4–9.6)
Female (*N* = 342)	12.1 (6.5–17.7)	860.4 (805.8–915.0)	9.6 (9.5–9.6)
*Age groups*			
55–64 years (*N* = 310)	7.0 (2.4–11.5)	988.4 (919.7–1057.2)	9.5 (9.4–9.6)
65–74 years (*N* = 227)	8.1 (2.8–13.4)	951.9 (860.5–1043.4)	9.6 (9.5–9.6)
75 + years (*N* = 133)	3.2 (− 1.0–7.4)	826.3 (738.0–914.7)	9.6 (9.5–9.7)
*Occupation*			
Employed (*N* = 99)	6.1 (− 1.3–13.6)	1120.4 (1010.2–1230.6)	9.5 (9.3–9.6)
Unemployed (*N* = 28)	4.3 (2.7–6.0)	877.3 (774.6–979.9)	9.5 (9.4–9.6)
Pensioner (*N* = 465)	5.5 (2.0–9.1)	954.5 (894.0–1015.0)	9.6 (9.5–9.6)
House-keeping (*N* = 93)	10.7 (− 0.3–21.6)	747.1 (674.5–819.8)	9.6 (9.5–9.7)
*Educational level*			
Low (*N* = 345)	7.3 (3.4–11.3)	926.6 (856.2–996.9)	9.6 (9.5–9.6)
Intermediate (*N* = 176)	5.7 (− 0.4–11.9)	965.9 (887.2–1044.7)	9.5 (9.4–9.5)
High (*N* = 164)	4.3 (− 0.9–9.4)	1020.5 (946.7–1094.4)	9.5 (9.4–9.6)
*Smoking*			
Daily smokers (*N* = 140)	7.1 (− 1.1–15.2)	929.6 (836.8–1022.4)	9.6 (9.5–9.7)
Occasional smokers (*N* = 23)	4.3 (− 6.5–15.1)	923.9 (761.2–1086.5)	9.4 (9.2–9.5)
Former smokers (*N* = 215)	3.2 (− 1.9–8.3)	1026.6 (932.3–1120.8)	9.5 (9.4–9.6)
Non smokers (*N* = 294)	9.0 (3.9–14.0)	907.5 (829.0–986.1)	9.6 (9.5–9.6)
*Urban level*			
Urban (*N* = 385)	5.9 (1.7–10.0)	931.1 (877.6–984.7)	9.5 (9.4–9.6)
Semi-urban (*N* = 150)	5.2 (0.2–10.1)	917.2 (830.1–1004.4)	9.5 (9.5–9.6)
Rural (*N* = 150)	8.5 (1.9–15.1)	995.3 (868.5–1122.2)	9.6 (9.5–9.7)
*BMI*			
< 25 (*N* = 98)	9.7 (– 0.6–19.9)	933.0 (819.8–1046.3)	9.5 (9.4–9.6)
25-29.9 (*N* = 278)	7.2 (3.1–11.3)	965.9 (893.3–1038.6)	9.5 (9.4–9.6)
≥ 30 (*N* = 275)	5.6 (0.1–11.1)	945.9 (866.4–1025.4)	9.6 (9.5–9.7)

Estimates account for sampling weights and complex survey design, along with non-response weights to ensure representativeness of the adult population. Mean values presented with 95% confidence intervals

**Table 4:** Univariable linear regression of factors potentially associated with blood calcium levels

Covariate	Coefficient (95% CI)	*p*-value
Non-food calcium intake (yes vs. no)	− 0.09 (− 0.45 to 0.26)	0.613
Sex (Female vs. Male)	0.08 (− 0.01 to 0.16)	0.081
*Age (years)*		
65–74 versus 54–64	0.08 (− 0.03 to 0.18)	0.145
75 + vs. 45–54	− 0.10 (− 0.01 to 0.22)	0.080
*Community setting*		
Semi-urban versus Urban	0.05 (− 0.06 to 0.16)	0.460
Rural versus Urban	0.10 (− 0.05 to 0.25)	0.210
*Smoking status*		
Occasional versus Current smokers	− 0.20 (− 0.37 to − 0.03)	0.019
Former versus Current smokers	− 0.04 (− 0.17 to 0.88)	0.516
Non- versus Current smokers	0.01 (− 0.11 to 0.13)	0.871
*Occupation*		
Unemployed versus Employed	0.04 (− 0.13 to 0.20)	0.653
Pensioner versus Employed	0.10 (− 0.04 to 0.24)	0.145
House-keeping versus Employed	0.15 (− 0.01 to 0.31)	0.074
*Educational level*		
F versus Low	− 0.11 (− 0.22 to 0.02)	0.053
High versus Low	− 0.06 (− 0.16 to 0.04)	0.233
*BMI*		
Overweight versus Normal weight	0.01 (− 0.12 to 0.14)	0.866
Obese versus Normal weight	0.08 (− 0.06 to 0.22)	0.261
Calcium food intake (adjusted to a 2000 kcal diet and per 1000 mg change)	− 0.011 (0.00 to 0.020)	0.024
Vitamin D Blood levels (per 1000 ng/ml change)	3.02 (− 1.43 to 9.08)	0.152

Coefficients represent the mean change in blood calcium concentration for each covariate category. Estimates account for sampling weights and complex survey design

## Discussion

Vitamin D and calcium deficiency have been associated with numerous health outcomes [3], and vitamin D deficiency in particular has become the most common nutritional deficiency worldwide [18, 46]. Our findings indicate that vitamin D insufficiency and deficiency were highly prevalent in this older Greek population, with women and the oldest adults exhibiting the poorest results. Women had lower serum vitamin D despite higher supplement use, while supplement intake emerged as the strongest determinant of vitamin D levels. Age showed a nonlinear association with vitamin D status, while dietary intake did not predict serum concentrations. For calcium, women consumed less dietary calcium and were the only supplement users, yet sex remained the sole independent predictor of serum calcium levels. Overall, vitamin D status in this cohort was driven mainly by supplement use, sex, and age rather than diet.

Concerning vitamin D, we found that 6.8% of Greeks aged 54 and over used vitamin D supplements (1.2% of men vs. 11.6% of women), while the mean dietary intake was 2.1 µg/day. According to EFSA average intake (AI) for Vitamin D is considered 15 µg/day. Considering previous findings indicating that dietary vitamin D intake in Europe ranged between 2.5 µg/day (adults 65 + in France) and 7.1 µg/day (men 65 + in Sweden) [37], our results suggest that the habitual diet of older adults in Greece is poor in vitamin D.

Mean vitamin D levels were 22.7 ng/mL, with 47.5% of participants presenting vitamin D deficiency (< 20 ng/mL). A previously published review reported that 35–75% of adults from Mediterranean countries have serum 25(OH)D concentrations < 20 ng/mL, with Greek adults averaging 54% in this range [29]. A more recent Greek study found 64.8% of participants had either deficient or insufficient vitamin D concentrations (< 20 ng/mL) [7]. In line with these previous reports, we found a high percentage of Greeks having vitamin D deficiency. Despite higher levels of sunlight exposure in the Mediterranean region, previous studies indicate that vitamin D status tend to be higher in northern regions of Europe [27], a difference often attributed to a diet rich in vitamin D and specifically to fish liver oil consumption [4], as well as the fortification in foods like milk, milk products and margarines [22].

Sex differences in serum vitamin D levels were evident in our sample. Men exhibited higher mean concentrations than women (24.6 vs. 21.1 ng/mL), while vitamin D deficiency was observed in 39.4% of men and 54.3% of women. Thus, despite the fact that drugs and supplements do improve vitamin D levels and that women use them more often compared to men, they still have lower vitamin D levels.

Although some studies report higher vitamin D serum levels in women compared to men [45], most authors agree that vitamin’s D deficiency is more prevalent among women [26]. Higher levels of estrogens promote the activation of 25(OH)D (the storage form) to 1,25(OH)_2_D (the active form). Consequently, the estrogens’ decline leads to vitamin D deficiency [14]. It has also been suggested that higher testosterone is also associated with higher vitamin D [50]. Besides hormonal differences, some lifestyle differences, such as clothing or occupational exposure to sunlight, may further contribute to sex differences in vitamin D status [24, 49].

Individuals aged 65–74 reported higher vitamin D intake and presented higher vitamin serum levels compared to 54–64 and ≥ 75 years old, suggesting individual decision or compliance to targeted interventions (e.g. public health messaging or clinical guidance) may be effective in maintaining vitamin D status [44]. An inverse U-shaped relationship between dietary vitamin D intake and age was observed, with intake increasing up to midlife and declining in older age.

Obesity was associated with higher intake but lower vitamin D blood levels. This apparent paradox is likely explained by sequestration in adipose tissue and reduced bioavailability [12, 13].

Regarding calcium, mean dietary intake among older HYDRIA participants was 948.1 mg/day. This level is below the NIH’s recommended range of 1000–1200 mg/day and the WHO expert advice for older adults, which can be as high as 1300 mg/day but it exceeds the EFSA Average Requirement of 750 mg/day [35, 36, 48, 10]. Women in our sample had lower mean intake levels than the Population Reference Intake (PRI) established by EFSA (950 mg/dl) [10]. Women’s mean calcium intake remained slightly below the EFSA Population Reference Intake (PRI) of 950 mg/day. Our estimates are slightly higher than the previously reported average calcium intake in Greece, estimated to be 882.4 mg/day in men and 738.7 mg/day in women 40–60 years old and 760.1 mg/day in women 50–75 years old [30]. In the EPIC study, the estimated values were higher, 1190 mg/day in men (from 1249 in 35–44 years old to 1085 in 65–74 years, differences non-significant) and 895 mg/day in women (from 949 in 45–54 years old to 840 in 65–74 years, differences non-significant) [47].

Globally, among 74 countries with available data, dietary calcium intake ranged from 175 mg/day (Nepal) to 1233 mg/day (Iceland). Most countries in South, East, and Southeast Asia consume less than 400–500 mg/day [27], as, for example in South Korea, where the average calcium intakes are estimated to be low (~ 485 mg/day) [23]. On the other hand, Northern European countries have intake levels exceeding 1000 mg/day [27]. For example in Iceland, where calcium intakes are higher, mean calcium intake is over 1000 mg/day [47]. In our study, average serum calcium was 9.6 mg/dL (9.5 in men and 9.6 in women). A recent study in Iran reported similar levels (mean 9.36 mg/dL), noting that in younger age men had higher average serum levels compared to women in the same age, a difference that reversed after age 50, likely due to lifestyle and dietary changes linked to menopause [40]. In the same study a drop in serum calcium in 2013 was also observed, possibly linked to changes in nutritional habits during economic hardship.

In our sample, only women aged 54–74 years reported taking calcium supplements, reflecting increased concern about postmenopausal bone health. Despite this, dietary calcium intake differences between sexes were small. Although women had slightly higher serum calcium, this may not be clinically significant. Serum calcium levels appear to remain stable across demographics, likely due to tight physiological regulation through parathyroid hormone and vitamin D-mediated absorption. Women in our study report the use of supplements at a significantly higher percentage (13.4%) compared to Finnish women (5.9%), although this comparison maybe biased due to the food fortification policies in Scandinavian countries [1].

Further research is needed to explore the biological implications of vitamin D deficiency and evaluate population-level interventions.

## Supplementary Information

Below is the link to the electronic supplementary material.

Supplementary Material 1

Supplementary Material 2

## References

[1] Alaghehband FR, Lyytinen AT, Isanejad M, Kopra J, Kröger H, Rikkonen T (2025) Long-term consumption of liquid dairy products predicts lower fracture risk in aging women: a 25-year follow-up. Eur J Nutr 64(5):213. 10.1007/s00394-025-03709-7 40488877 10.1007/s00394-025-03709-7 PMC12149010

[2] Albani E, Petrou P (2020) A systematic review and meta-analysis of vitamin D and calcium in preventing osteoporotic fractures. Clin Rheumatol 39(12):3571–3579. 10.1007/s10067-020-05122-3 32447604 10.1007/s10067-020-05122-3

[3] Berger C, Greene-Finestone LS, Langsetmo L, Kreiger N, Joseph L, Kovacs CS, Richards JB, Hidiroglou N, Sarafin K, Davison KS, Adachi JD, Brown J, Hanley DA, Prior JC, Goltzman D (2012) Temporal trends and determinants of longitudinal change in 25-hydroxyvitamin D and parathyroid hormone levels. J Bone Miner Res 27(6):1381–1389. 10.1002/jbmr.1587. the CaMos Research Group 22407786 10.1002/jbmr.1587 PMC5101062

[4] Brustad M, Sandanger T, Aksnes L, Lund E (2004) Vitamin D status in a rural population of northern Norway with high fish liver consumption. Public Health Nutr 7(6):783–789. 10.1079/PHN2004605 15369617 10.1079/phn2004605

[5] Chiang C, Ismaeel A, Griffis RB, Weems S (2017) Effects of vitamin D supplementation on muscle strength in athletes: a systematic review. J Strength Conditioning Res 31(2):566–574. 10.1519/JSC.0000000000001518 10.1519/JSC.0000000000001518 27379960

[6] DeLuca HF (2004) Overview of general physiologic features and functions of vitamin D. Am J Clin Nutr 80(6):1689S–1696S. 10.1093/ajcn/80.6.1689S 15585789 10.1093/ajcn/80.6.1689S

[7] Dimakopoulos I, Magriplis E, Mitsopoulou A-V, Karageorgou D, Bakogianni I, Micha R, Michas G, Chourdakis M, Ntouroupi T, Tsaniklidou S-M, Argyri K, Panagiotakos DB, Zampelas A, Fappa E, Theodoraki E-M, Trichia E, Sialvera T-E, Varytimiadi A, Spyreli E, Roma E (2019) Association of serum vitamin D status with dietary intake and sun exposure in adults. Clin Nutr ESPEN 34:23–31. 10.1016/j.clnesp.2019.09.008 31677707 10.1016/j.clnesp.2019.09.008

[8] Dusso AS, Brown AJ, Slatopolsky E (2005) Vitamin D. Am J Physiology-Renal Physiol 289(1):F8–F28. 10.1152/ajprenal.00336.2004 10.1152/ajprenal.00336.2004 15951480

[9] European Food Safety Authority (2014) Guidance on the EU Menu methodology. EFSA J 12(12). 10.2903/j.efsa.2014.3944

[10] European Food Safety Authority (EFSA) (2017) Dietary Reference Values for nutrients Summary report. EFSA Supporting Publications 14(12). 10.2903/sp.efsa.2017.e15121

[11] European Health Examination Survey (2016) EHES manuals: general guidelines and examination protocols (Parts A–F). National Institute for Health and Welfare (THL). https://ehes.info/manuals.htm

[12] Farhud DD, Zarif-Yeganeh M, Mehrabi A, Afshari A-R, Rokni MB, Majidi K, Jalali M, Zargar AAA, Sarafnejad A, Sadeghipour HR, Zokaei S, Khosravi F, Jalali M, Khazeni M (2022) A retrospective study of serum calcium status in Tehran, Iran (105,128 Samples, from 2009–2018). Iran J Public Health. 10.18502/ijph.v51i4.9245 35936520 10.18502/ijph.v51i4.9245 PMC9288392

[13] for the International Osteoporosis Foundation Calcium Steering Committee, Balk EM, Adam GP, Langberg VN, Earley A, Clark P, Ebeling PR, Mithal A, Rizzoli R, Zerbini CAF, Pierroz DD, Dawson-Hughes B (2017) Global dietary calcium intake among adults: a systematic review. Osteoporos Int 28(12):3315–3324. 10.1007/s00198-017-4230-x 29026938 10.1007/s00198-017-4230-x PMC5684325

[14] Gallagher JC, Sai A, Templin T, Smith L (2012) Dose response to vitamin D supplementation in postmenopausal women: a randomized trial. Ann Intern Med 156(6):425–437. 10.7326/0003-4819-156-6-201203200-00005 22431675 10.7326/0003-4819-156-6-201203200-00005

[15] Gomes F, Ashorn P, Askari S, Belizan JM, Boy E, Cormick G, Dickin KL, Driller-Colangelo AR, Fawzi W, Hofmeyr GJ, Humphrey J, Khadilkar A, Mandlik R, Neufeld LM, Palacios C, Roth DE, Shlisky J, Sudfeld CR, Weaver C, Bourassa MW (2022) Calcium supplementation for the prevention of hypertensive disorders of pregnancy: current evidence and programmatic considerations. Ann N Y Acad Sci 1510(1):52–67. 10.1111/nyas.14733 35000200 10.1111/nyas.14733 PMC9306576

[16] Habibi Ghahfarrokhi S, Mohammadian-Hafshejani A, Sherwin CMT, Heidari-Soureshjani S (2022) Relationship between serum vitamin D and hip fracture in the elderly: a systematic review and meta-analysis. J Bone Miner Metab 40(4):541–553. 10.1007/s00774-022-01333-7 35639176 10.1007/s00774-022-01333-7

[17] Holick MF (2007) Vitamin D deficiency. N Engl J Med 10.1056/NEJMra070553 17634462

[18] Holick MF (2017) The vitamin D deficiency pandemic: approaches for diagnosis, treatment and prevention. Reviews Endocr Metabolic Disorders 18(2):153–165. 10.1007/s11154-017-9424-1 10.1007/s11154-017-9424-1 28516265

[19] Holick MF, Binkley NC, Bischoff-Ferrari HA, Gordon CM, Hanley DA, Heaney RP, Murad MH, Weaver CM (2011) Evaluation, treatment, and prevention of vitamin D deficiency: an endocrine society clinical practice guideline. J Clin Endocrinol Metabolism 96(7):1911–1930. 10.1210/jc.2011-0385 10.1210/jc.2011-0385 21646368

[20] Holick MF, Chen TC (2008) Vitamin D deficiency: a worldwide problem with health consequences. Am J Clin Nutr 87(4):1080S–1086S. 10.1093/ajcn/87.4.1080S 18400738 10.1093/ajcn/87.4.1080S

[21] Ismailova A, White JH (2022) Vitamin D, infections and immunity. Rev Endocr Metabolic Disorders 23(2):265–277. 10.1007/s11154-021-09679-5 10.1007/s11154-021-09679-5 PMC8318777 34322844

[22] Itkonen ST, Andersen R, Björk AK, Brugård Konde Å, Eneroth H, Erkkola M, Holvik K, Madar AA, Meyer HE, Tetens I, Torfadóttir JE, Thórisdóttir B, Lamberg-Allardt CJE (2021) Vitamin D status and current policies to achieve adequate vitamin D intake in the Nordic countries. Scand J Public Health 49(6):616–627. 10.1177/1403494819896878 31916497 10.1177/1403494819896878

[23] Joo N-S, Dawson-Hughes B, Kim Y-S, Oh K, Yeum K-J (2013) Impact of calcium and vitamin D insufficiencies on serum parathyroid hormone and bone mineral density: analysis of the fourth and fifth Korea National Health and Nutrition Examination Survey (KNHANES IV-3, 2009 and KNHANES V-1, 2010). J Bone Miner Res 28(4):764–770. 10.1002/jbmr.1790 23045165 10.1002/jbmr.1790

[24] Kader S, Comaklı H, Tekindal MA (2019) Evaluation of serum Vitamin D levels according to gender and age at Karapınar city: a follow-up study from Turkey. Dubai Med J 2(4):141–145. 10.1159/000503899

[25] Kahwati LC, Weber RP, Pan H, Gourlay M, LeBlanc E, Coker-Schwimmer M, Viswanathan M (2018) Vitamin D, calcium, or combined supplementation for the primary prevention of fractures in community-dwelling adults: evidence report and systematic review for the US preventive services task force. JAMA 319(15):1600. 10.1001/jama.2017.2164029677308 10.1001/jama.2017.21640

[26] Liel Y, Shany S, Smirnoff P, Schwartz B (1999) Estrogen increases 1,25-Dihydroxyvitamin D receptors expression and bioresponse in the rat duodenal mucosa. Endocrinology 140(1):280–285. 10.1210/endo.140.1.6408. This study was supported, in part, by a grant from the Chief Scientist’s Office, the Ministry of Health of the State of Israel, and by a grant from the Faculty of Health Sciences, Ben-Gurion University of the Negev 9886836 10.1210/endo.140.1.6408

[27] Lips P (2007) Vitamin D status and nutrition in Europe and Asia. J Steroid Biochem Mol Biol 103(3–5):620–625. 10.1016/j.jsbmb.2006.12.076 17287117 10.1016/j.jsbmb.2006.12.076

[28] Maai N, Frank FA, Meuris A, Ferreira N (2025) Association between vitamin D and musculoskeletal injuries: a systematic review. Cureus. 10.7759/cureus.82495 40385928 10.7759/cureus.82495 PMC12085790

[29] Manios Y, Moschonis G, Lambrinou C-P, Tsoutsoulopoulou K, Binou P, Karachaliou A, Breidenassel C, Gonzalez-Gross M, Kiely M, Cashman KD (2018) A systematic review of vitamin D status in southern European countries. Eur J Nutr 57(6):2001–2036. 10.1007/s00394-017-1564-2 29090332 10.1007/s00394-017-1564-2

[30] Manios Y, Moschonis G, Mavrogianni C, Bos R, Singh-Povel C (2014) Micronutrient intakes among children and adults in Greece: the role of age, sex and socio-economic status. Nutrients 6(10):4073–4092. 10.3390/nu6104073 25285410 10.3390/nu6104073 PMC4210908

[31] Martimianaki G, Naska A, Papatesta ME, Peppa E, Orfanos P, Trichopoulou A (2022) Methods and introductory results of the Greek national health and nutrition survey—HYDRIA. Epidemiol Biostatistics Public Health 15(2). 10.2427/12914

[32] Martimianaki G, Peppa E, Valanou E, Papatesta EM, Klinaki E, Trichopoulou A (2022) Today’s mediterranean diet in Greece: findings from the National Health and Nutrition Survey—HYDRIA (2013–2014). Nutrients 14(6):1193. 10.3390/nu14061193 35334847 10.3390/nu14061193 PMC8949101

[33] Migliorini F, Maffulli N, Colarossi G, Filippelli A, Memminger M, Conti V (2025) Vitamin D and calcium supplementation in women undergoing pharmacological management for postmenopausal osteoporosis: a level I of evidence systematic review. Eur J Med Res 30(1):170. 10.1186/s40001-025-02412-x 40087804 10.1186/s40001-025-02412-x PMC11907966

[34] Mirza AMW, Almansouri NE, Muslim MF, Basheer T, Uppalapati SV, Lakra S, Fatima H, Adhnon A, Filho IW, Mahmood R, Kumar M, Kandel K, Ayyan M (2024) Effect of vitamin D supplementation on cardiovascular outcomes: an updated meta-analysis of RCTs. Annals Med Surg 86(11):6665–6672. 10.1097/MS9.0000000000002458 10.1097/MS9.0000000000002458 PMC11543226 39525782

[35] National Health Service (NHS). (n.d.) How much calcium do I need? NHS. Retrieved 3 June 2025 from https://www.nhs.uk/conditions/vitamins-and-minerals/calcium/. Access 03 August 2020

[36] Office of Dietary Supplements, National Institutes of Health. (n.d.) Calcium: fact sheet for health professionals. National Institutes of Health. Retrieved 3 (2025) June from https://ods.od.nih.gov/factsheets/Calcium-HealthProfessional/. Access Date 24 July 2024

[37] Ovesen L, Andersen R, Jakobsen J (2003) Geographical differences in vitamin D status, with particular reference to European countries. Proc Nutr Soc 62(4):813–821. 10.1079/PNS2003297 15018480 10.1079/PNS2003297

[38] Pilz S, Zittermann A, Obeid R, Hahn A, Pludowski P, Trummer C, Lerchbaum E, Pérez-López FR, Karras SN, März W (2018) The role of vitamin D in fertility and during pregnancy and lactation: a review of clinical data. Int J Environ Res Public Health 15(10):2241. 10.3390/ijerph15102241 30322097 10.3390/ijerph15102241 PMC6210343

[39] Prentice A (2008) Vitamin D deficiency: a global perspective: Nutrition Reviews©, Vol. 66, No. s2. Nutrition Reviews, 66:S153–S164. 10.1111/j.1753-4887.2008.00100.x 10.1111/j.1753-4887.2008.00100.x 18844843

[40] Rezaei OM, Sharifi F, Moodi M, Zarban A, Tahergorabi R, Tahergorabi Z (2023) The prevalence and determinants of vitamin D status among older adults: data from a longitudinal aging study. Int J Prev Med 14(1). 10.4103/ijpvm.ijpvm_366_21 10.4103/ijpvm.ijpvm_366_21 PMC10080568 37033285

[41] Roy N, M (2021) Impact of vitamin D on neurocognitive function in dementia, depression, schizophrenia and ADHD. Front Biosci 26(3):566–611. 10.2741/4908 10.2741/4908 33049684

[42] Shlisky J, Mandlik R, Askari S, Abrams S, Belizan JM, Bourassa MW, Cormick G, Driller-Colangelo A, Gomes F, Khadilkar A, Owino V, Pettifor JM, Rana ZH, Roth DE, Weaver C (2022) Calcium deficiency worldwide: prevalence of inadequate intakes and associated health outcomes. Ann N Y Acad Sci 1512(1):10–28. 10.1111/nyas.14758 35247225 10.1111/nyas.14758 PMC9311836

[43] Steingrimsdottir L (2005) Relationship between serum parathyroid hormone levels, vitamin D sufficiency, and calcium intake. JAMA 294(18):2336. 10.1001/jama.294.18.2336 16278362 10.1001/jama.294.18.2336

[44] Subramanian A, Burrowes HB, Rumph JT, Wilkerson J, Jackson CL, Jukic AMZ (2024) Vitamin D levels in the United States: temporal trends (2011–2018) and contemporary associations with sociodemographic characteristics (2017–2018). Nutrients 16(19):3414. 10.3390/nu16193414 39408379 10.3390/nu16193414 PMC11478591

[45] Vallejo MS, Blümel JE, Arteaga E, Aedo S, Tapia V, Araos A, Sciaraffia C, Castelo-Branco C (2020) Gender differences in the prevalence of vitamin D deficiency in a southern Latin American country: a pilot study. Climacteric 23(4):410–416. 10.1080/13697137.2020.1752171 32367772 10.1080/13697137.2020.1752171

[46] Van Schoor N, Lips P (2017) Global overview of vitamin D status. Endocrinol Metab Clin North Am 46(4):845–870. 10.1016/j.ecl.2017.07.002 29080639 10.1016/j.ecl.2017.07.002

[47] Welch AA, Fransen H, Jenab M, Boutron-Ruault MC, Tumino R, Agnoli C, Ericson U, Johansson I, Ferrari P, Engeset D, Lund E, Lentjes M, Key T, Touvier M, Niravong M, Larrañaga N, Rodríguez L, Ocké MC, Peeters PHM, Bingham S (2009) Variation in intakes of calcium, phosphorus, magnesium, iron and potassium in 10 countries in the European prospective investigation into cancer and nutrition study. Eur J Clin Nutr 63(S4):S101–S121. 10.1038/ejcn.2009.77 19888269 10.1038/ejcn.2009.77

[48] World Health Organization (2004) Vitamin and Mineral Requirements in Human Nutrition (2nd edn). World Health Organ. https://www.who.int/publications/i/item/9241546123

[49] Yan X, Zhang N, Cheng S, Wang Z, Qin Y (2019) Gender differences in vitamin D status in China. Med Sci Monit 25:7094–7099. 10.12659/MSM.916326 31541605 10.12659/MSM.916326 PMC6767943

[50] Zhao D, Ouyang P, De Boer IH, Lutsey PL, Farag YMK, Guallar E, Siscovick DS, Post WS, Kalyani RR, Billups KL, Michos ED (2017) Serum vitamin D and sex hormones levels in men and women: the multi-ethnic study of atherosclerosis (MESA). Maturitas 96:95–102. 10.1016/j.maturitas.2016.11.017 28041602 10.1016/j.maturitas.2016.11.017 PMC5218632

