# Supplementary material for: Vitamin D and calcium status in HYDRIA, Greece: associations with dietary and supplemental sources
Source: Eur J Nutr. 2026 Apr 10;65(3):114. doi: 10.1007/s00394-026-03962-4 (PMC13068735; doi:10.1007/s00394-026-03962-4)
Supplement: Supplementary file 1 — Supplementary Material 1 [file 394_2026_3962_MOESM1_ESM.docx]

**Table 2.** Univariable linear regression of factors potentially associated with blood vitamin D levels.

| **Covariate** | **Coefficient (95% CI)** | **p-value** |
| --- | --- | --- |
| **Non-food vitamin D intake** (yes vs. no) | 6.81 (4.27 to 9.34) | <0.001 |
| **Sex** (Female vs. Male) | -3.44 (-5.95 to -0.93) | 0.008 |
| **Age** (years) | 1.28 (-0.32 to 2.88) | 0.116 |
| **Squared Age** (years^2^) | -0.01 ( -0.02 to -0.00) | 0.074 |
| **Community Setting** |  |  |
| semi-urban vs. urban | -0.07 (-3.09 to 2.95) | 0.963 |
| rural vs. urban | -0.84 (-3.48 to 1.80) | 0.527 |
| **Smoking status** |  |  |
| Occasional vs. current smokers | -1.13 (-5.34 to 3.07) | 0.593 |
| Former vs. current smokers | 0.68 (-2.51 to 3.88) | 0.672 |
| Non- vs. current smokers | -1.61 (-4.74 to 1.52) | 0.310 |
| **Occupation** |  |  |
| Unemployed vs. Employed | 4.86 (-0.03 to 9.74) | 0.051 |
| Pensioner vs. Employed | 0.95 (-2.14 to 4.03) | 0.545 |
| House-keeping vs. Employed | -2.18 (-6.01 to 1.66) | 0.263 |
| **Educational level** |  |  |
| Intermediate vs. low | 2.53 (0.11-4.94) | 0.041 |
| High vs. low | 3.91 (1.64-6.17) | 0.001 |
| **BMI** |  |  |
| Overweight vs. Normal weight | -0.78 (-4.50 to 2.94) | 0.679 |
| Obese vs. Normal weight | -3.43 (-6.90 to 0.03) | 0.052 |
| **Vitamin D food intake** (per 1mg change) | 0.13 (-0.15 to 0.41) | 0.371 |

*Coefficients represent the mean change in vitamin D concentration for each covariate category change. Estimates account for sampling weights and complex survey design to ensure representativeness of the adult population.*
