# Supplementary material for: Vitamin D and calcium status in HYDRIA, Greece: associations with dietary and supplemental sources
Source: Eur J Nutr. 2026 Apr 10;65(3):114. doi: 10.1007/s00394-026-03962-4 (PMC13068735; doi:10.1007/s00394-026-03962-4)
Supplement: Supplementary file 2 — Supplementary Material 2 [file 394_2026_3962_MOESM2_ESM.docx]

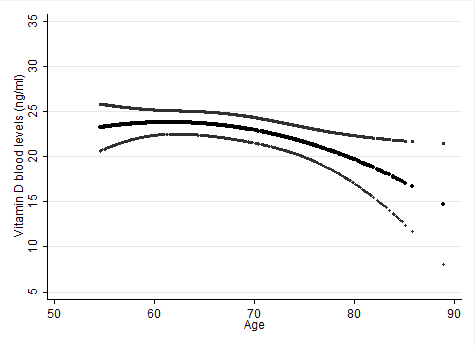


Supplementary Figure 1. Vitamin D blood levels and corresponding 95% Confidence Interval of all study participants. Predictions come from an unadjusted linear regression model.


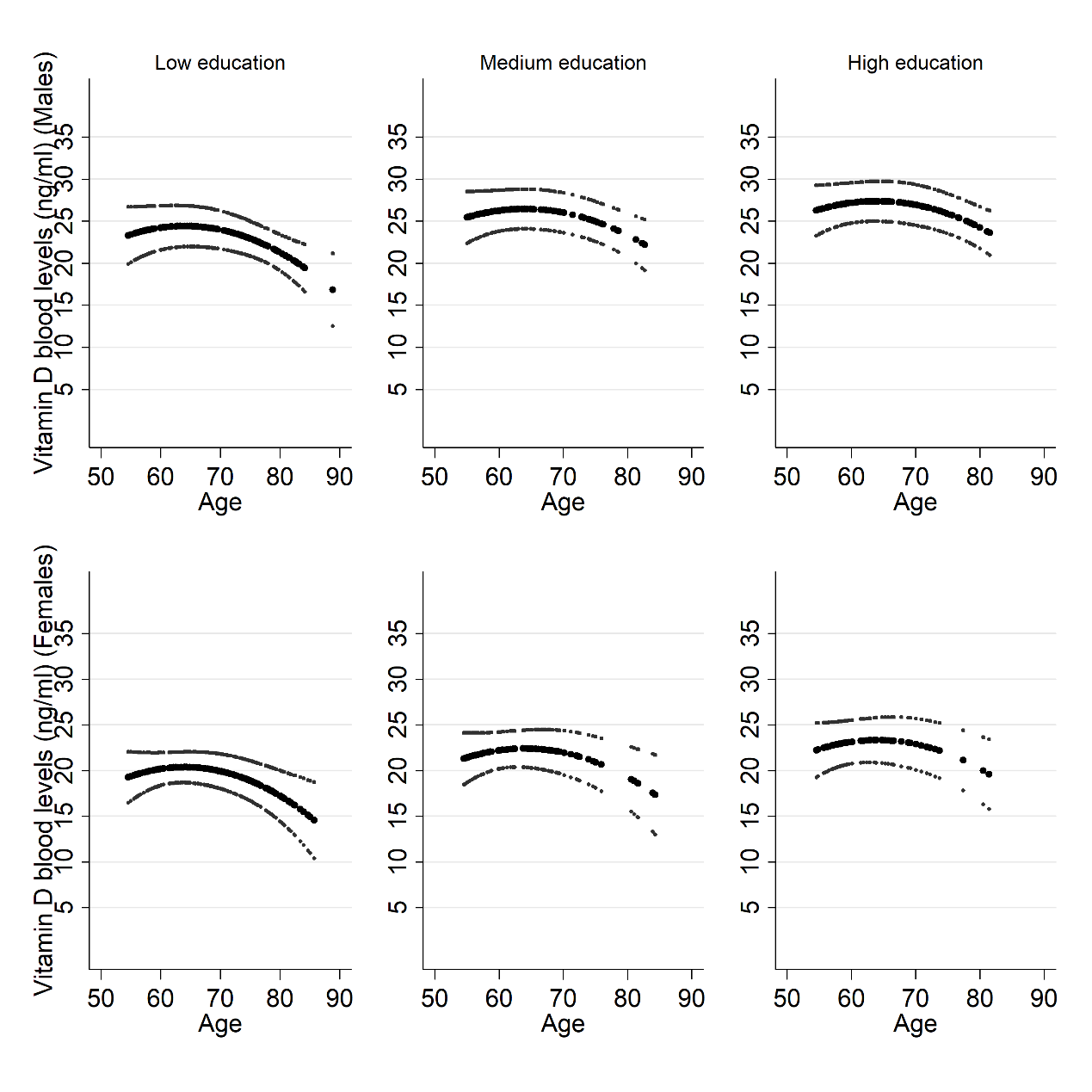


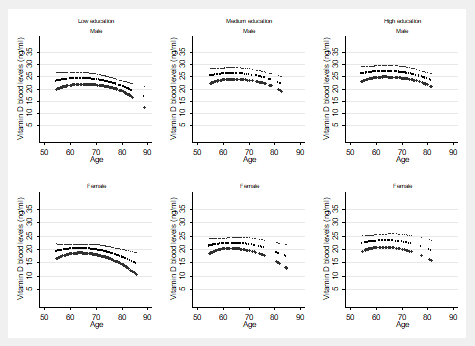


Supplementary Figure 2. Vitamin D blood levels and corresponding 95% Confidence Interval in men (upper panel) and women (lower panel) non-supplement users. The three columns correspond to low, medium and high educational levels. Predictions come from an unadjusted linear regression model.
